# A Memristor SPICE Model Accounting for Synaptic Activity Dependence

**DOI:** 10.1371/journal.pone.0120506

**Published:** 2015-03-18

**Authors:** Qingjiang Li, Alexander Serb, Themistoklis Prodromakis, Hui Xu

**Affiliations:** 1 College of Electronic Science and Engineering, National University of Defense Technology, Changsha, Hunan, China; 2 Nano Group, Southampton Nanofabrication Centre, Department of Electronic and Computer Science, University of Southampton, Southampton, Hampshire, United Kingdom; Georgia State University, UNITED STATES

## Abstract

In this work, we propose a new memristor SPICE model that accounts for the typical synaptic characteristics that have been previously demonstrated with practical memristive devices. We show that this model could account for both volatile and non-volatile memristance changes under distinct stimuli. We then demonstrate that our model is capable of supporting typical STDP with simple non-overlapping digital pulse pairs. Finally, we investigate the capability of our model to simulate the activity dependence dynamics of synaptic modification and present simulated results that are in excellent agreement with biological results.

## Introduction

Recently, it has been demonstrated that the memristor is a promising candidate for implementing single device based artificial synapses [1–4] as its memristance depends not only on instantaneous external inputs but also on its past history [5]. Furthermore, the capability of single memristors to exhibit key ‘synapse-like’ behaviors such as long-term potentiation (LTP), long-term depression (LTD), and even spike-timing-dependent plasticity (STDP) have been experimentally demonstrated in solid-state memristive devices [1–4].

What is missing is an empirical model that is capable of capturing the experimentally observed synaptic behaviors. The availability of such model would greatly facilitate the development of memristor-based neuromorphic applications [6], [7]. Current established memristor models [8], [9] only feature non-volatile internal state variables [3], [10]. As a result, they can only partially capture the rich variety of observed ‘synapse-like’ characteristics when biased with specifically designed overlapping spike-like waveforms [11], which requires additional complex circuits to generate.

We have previously proposed a memristor model to account for the volatile memristance dynamics [3]. Here, we further improve this model by incorporating a synaptic activity dependence module. Moreover, it is worth highlighting that our new model can simulate both typical STDP [12], [13] and LTD/LTP dependence on spike-pair frequency [14] within the context of simple, non-overlapping digital pulse pair stimulation. This has significant ramifications for memristor-based neuromorphic applications as it enables a reduction in circuitry complexity and power dissipation.

In this paper, we first show that our model, with all extra features, can still produce the memristor signature I-V pinched-hysteresis loop. We then show that the model could account for both volatile and non-volatile memristance changes in response to stimuli with appropriately defined amplitude and width. Then, we exploit the model for simulating pair-based STDP behavior and exploring its dependence on the input pulse width, amplitude and model parameters. Finally, we monitored the overall memristance change after the application of a bipolar pulse-pair train as a function of the pulse-pair frequency, with simulation results correlating well with synaptic activity dependence (i.e. the phenomenon of synaptic modification dependence on overall pre/post spike frequency) as observed in biological synapses [14].

## Activity Dependence Model

**Fig 1:**
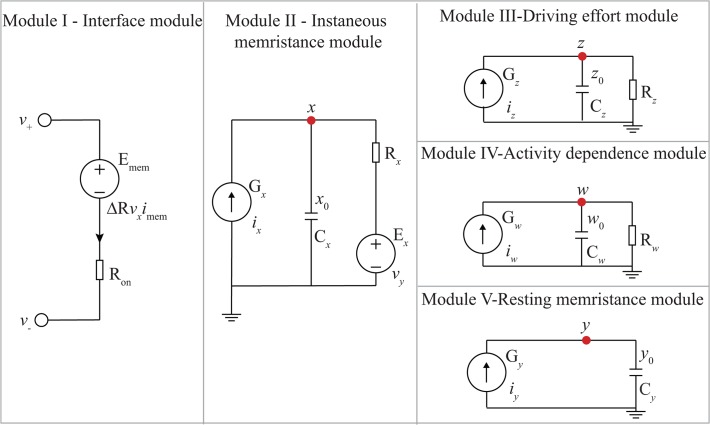
Schematic of the proposed memristor SPICE model. Parameters utilized in simulations are: *R*_*on*_ = 1Ω, *R*_*off*_ = 100kΩ, *R*_*init*_ = 5kΩ, *ε* = 10^6^, *C*_*x*_ = 5mF, *R*_*x*_ = 1Ω, *C*_*y*_ = 0.15F, *C*_*z*_ = 1F, *R*_*z*_ = 3mΩ, *C*_*w*_ = 1F, *R*_*w*_ = 0.35Ω, *B*_+_ = −*B*_−_ = 0.35nV, *k* = 0.33e^10^, *α* = 0.706, *β* = 1e^8^, *γ* = 2, *p* = 0.5, *j* = 2 and *m* = 0.62. The initial condition of four internal variables are set as *x*_0_ = *y*_0_ = (*R*_*off*_ − *R*_*init*_) / (*R*_*off*_ − *R*_*on*_), and *z*_0_ = *w*_0_ = 0.

The model examined in this work consists of five modules (Fig. 1), which are easy to implement in a SPICE environment.

### Module I

Interface: In module I, the memristance of the modelled device as seen by external circuitry is generated by a series combination of the fixed *R*_*on*_ resistor and a voltage-controlled voltage source E_mem_, whose terminal voltage is controlled by node potential *v*_*x*_ ∊ [0,1] of the module II as:

$$R_{mem}(v_{x})=R_{on}+\Delta Rv_{x},\;\Delta R=R_{off}-R_{on}$$ (1)

### Module II

Instantaneous memristance: The purpose of modules II-V is only to solve differential equations and thus have no physical equivalent in the system. In contrast to Biolek’s memristor model [8], module II features a leak path to account for volatile dynamics and specified by the values of *C*_*x*_ and *R*_*x*_. In the absence of external stimuli, the instantaneous memristance decays exponentially to the resting memristance (defined by *v*_*y*_ in module V) as:

$$C_{x}\frac{dv_{x}}{dt}=-\frac{v_{x}-v_{y}}{R_{x}}+i_{x},\;i_{x}=\varepsilon i_{mem}f\left(v_{x}\right)$$ (2)

where *f*(*v*_*x*_) is a rectangular window function defined in [9] that confines the instantaneous memristance *R*_*mem*_ within the [M_min_,M_max_] interval. The window function takes the value *f*(M) = 1 in the interval M ∊ (0,1), but for M ∊ {0,1}, *v*_*x*_ is restricted to changing towards the inside of the allowed memristance interval. *ε* is a constant that is inherited from Biolek's *μ*_*v*_/(2*D* ^2^) constant parameter [8] and is effectively a `lumped constant' that introduces the effects of device geometry and fabrication into the system of equations.

### Module III

Driving effort: *C*_*z*_ and *R*_*z*_ form a leaky integrator with state variable *v*_*z*_ integrating all external driving efforts, in this case the external driving voltage across the memristor *v*_*mem*_ as:

$$C_{z}\frac{dv_{z}}{dt}=i_{z}-\frac{v_{z}}{R_{z}}=\frac{v_{mem}}{0.5•(R_{on}+R_{Roff})}-\frac{v_{z}}{R_{z}}$$ (3)

### Module IV

Activity dependence: In practical memristors, Joule heating is expected to significantly affect memristor dynamics as it would determine the annihilation of conductive percolation channels within active cores [15], [16]. Thus, we included within our model a new, activity dependence module to account for the influence of activity-dependent thermal accumulation on memristor dynamics [16]. This is implemented by introducing state variable *v*_*w*_, which integrates the absolute power dissipation via a leaky progress defined by *C*_*w*_ and *R*_*w*_ as:

$$C_{w}\frac{dv_{w}}{dt}=i_{w}-\frac{v_{w}}{R_{w}}=\left|i_{mem}•v_{mem}\right|-\frac{v_{w}}{R_{w}}$$ (4)

### Module V

Resting memristance: Purpose-built functions allow the driving variable *v*_*z*_ and activity dependence variable *v*_*w*_ plug into the system and influence the resting memristance as:

$$C_{y}\frac{dv_{y}}{dt}=i_{y}=f\left(v_{y}\right)\phi \left(v_{z},{\text{B}}_{+},{\text{B}}_{-}\right)h\left(v_{w}\right)$$ (5)

Function *f*(*v*_*y*_) is the rectangular window function that restricts *v*_*y*_ within the maximum/minimum memristance limits and is also used in module II.

Function *ϕ* determines the dependence of resting memristance on the driving effort being applied to the device. In this manuscript, we modified Pershin’s threshold window function [9] to allow the definition of two distinct operating regions. Specifically, when the effort variable *v*_*z*_ lies in within the positive and negative bipolar thresholds *B*_+_ and *B*_−_, the memristor would be operated in ‘sub-threshold mode’ and the resting memristance would not change at all. In contrast, when *v*_*z*_ is above *B*_+_ or below *B*_−_, the memristor would operate in bipolar mode where the values of *ϕ* would be in proportion to the amount by which the effort exceeds the bipolar threshold in both directions. Function *ϕ* is given by:

$$\phi \left(v_{z},B_{+},B_{-}\right)=\{\begin{array}{cc} k{\left(v_{z}-B_{\pm }\right)}^{m} & \text{if}\;v_{z}\in \{[-\infty ,B_{-}),(B_{+},+\infty ]\}\\ 0 & \text{if}\;v_{z}\in [B_{-},B_{+}] \end{array}$$ (6)

Where *k* is a scaling constant and *m* is the factor that determines the specific curve shape.

Function *h* determines the influence of the activity dependence variable (*v*_*w*_) on resting memristance. In this manuscript, the specific form of function *h* is set up based on activity dependence data of biological synapses as per [14]. We constructed a two-valued function that depends on the sign of the driving effort variable *v*_*z*_, as:

$$h\left(v_{w}\right)=\{\begin{array}{ll} \alpha •({\left(\beta v_{w}\right)}^{p}+1) & v_{z}>0\\ \;1-\gamma {\text{(}\beta v_{w}\text{)}}^{j} & v_{z}\leq 0 \end{array}$$ (7)

Where *a*, *β*, *γ* are the scaling constants, while *p* and *j* set up the specific curve shape.

Finally, it is worth stressing that in our model, windowing, drivability and activity dependence operate on *v*_*y*_ in a multiplicative fashion in order to render the system less complicated whilst still allowing the exhibition of important biomimetic behavior.

## Results and Discussion

### 3.1. Memristive I-V response

**Fig 2:**
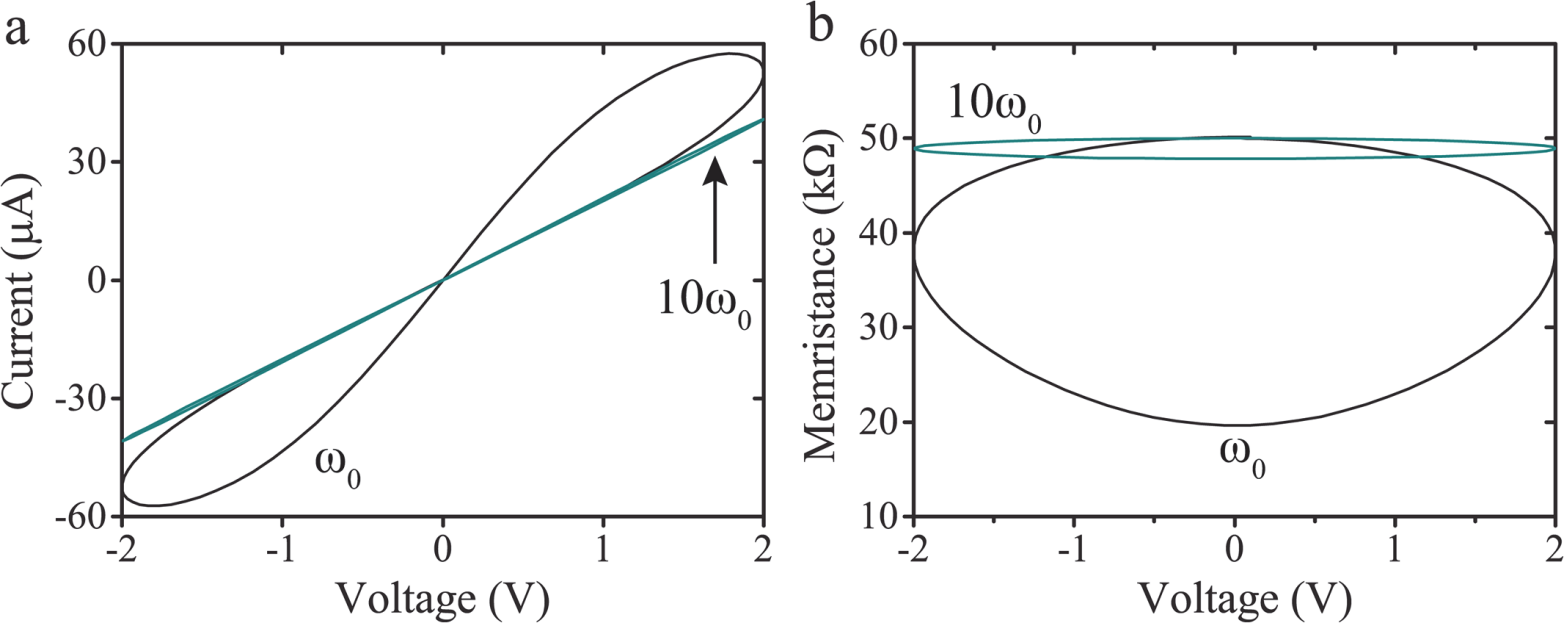
Memristor model behaviour. (a) Simulated pinched hysteresis I-V responses at frequencies of ω_0_ and 10ω_0_. (b) Corresponding memristance as a function of applied voltage.

To verify the memristive characteristics of our proposed model, we employed a sinusoid stimulus at two different frequencies. The attained pinched hysteresis I-V curves shown in Fig. 2A are the typical fingerprint of bipolar resistive switching [5], with the loop area shrinking at higher frequency (10ω_0_). The corresponding resistance response is illustrated in Fig. 2B with results demonstrating that the prominence of resistive switching is significantly reduced at higher frequencies, which correlates with memristor theory.

### 3.2. Volatile and non-volatile memristance dynamics

One of the typical characteristics of memristive devices is the non-volatile resistance change under external stimuli [5], [17]. However, this may not always apply for practical devices. Recently, it has been experimentally demonstrated that under weak stimulus, memristance could be driven into a temporary state and then decay back to the original level [3], [18]. Thus, in our proposed model, both volatile and non-volatile resistance dynamics have been taken into consideration.

**Fig 3:**
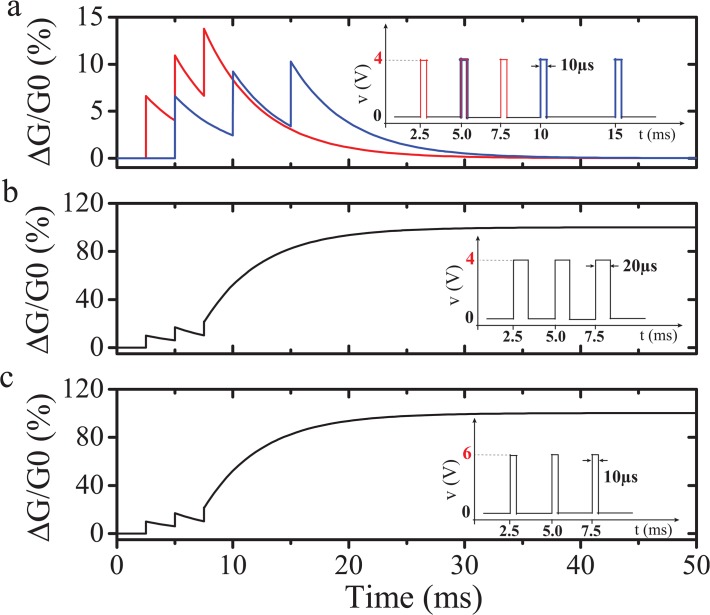
(a) Modelled normalized volatile conductance (*G*(*t*) = 1 / *R*_*mem*_(*t*)) dynamics in response to three consecutive pulses possessing varying inter-pulse intervals: 2.5ms (red line) and 5ms (blue line), respectively. For both cases, pulse amplitude and width were fixed at 4V, 10μs and the system remains fully volatile. (b) Transition from volatile to non-volatile dynamics due to a change in pulse width (10 μs to 20μs). (c) Transition from volatile to non-volatile dynamics due to a change in pulse amplitude (4V to 6V).

Initially, we explore the volatile response of the model by applying relatively weak stimuli. Fig. 3A illustrates the normalized volatile conductance change in response to three positive pulses (4V, 10μs) and its dependence on pulse intervals. Conductance initially increases (during each pulse stimulus) and subsequently decays towards its original value (between stimuli). Moreover, the volatile dynamics are sensitive to the employed pulse interval, as we have experimentally demonstrated previously [3].

We further explore the model to evaluate non-volatile memristance dynamics under stronger driving efforts. In specific, the stronger driving efforts were implemented via increasing either pulse width (10 μs to 20μs) or amplitude (4V to 6V) with results demonstrated in Fig. 3B and 3C, respectively. In both cases, the volatile dynamics now transfer to non-volatile change at the 3^rd^ pulse event. This phenomenon stems from the different mechanisms that produce the rates of change of volatile variable *v*_*x*_ and non-volatile variable *v*_*y*_. In case of weak stimuli, the rate of change of *v*_*x*_ is relatively larger and that accentuates the volatile dynamics. In contrast, when biased with stronger stimuli, the elevated rate of change of non-volatile variable *v*_*y*_ would outweigh that of *v*_*x*_ and the contribution of the volatile decay circuit (*C*_*x*_ and *R*_*x*_) would thus become insignificant. In this case, the model resembles a Biolek-type, fully non-volatile SPICE model more closely.

### 3.3. Spike-timing-dependent plasticity

**Fig 4:**
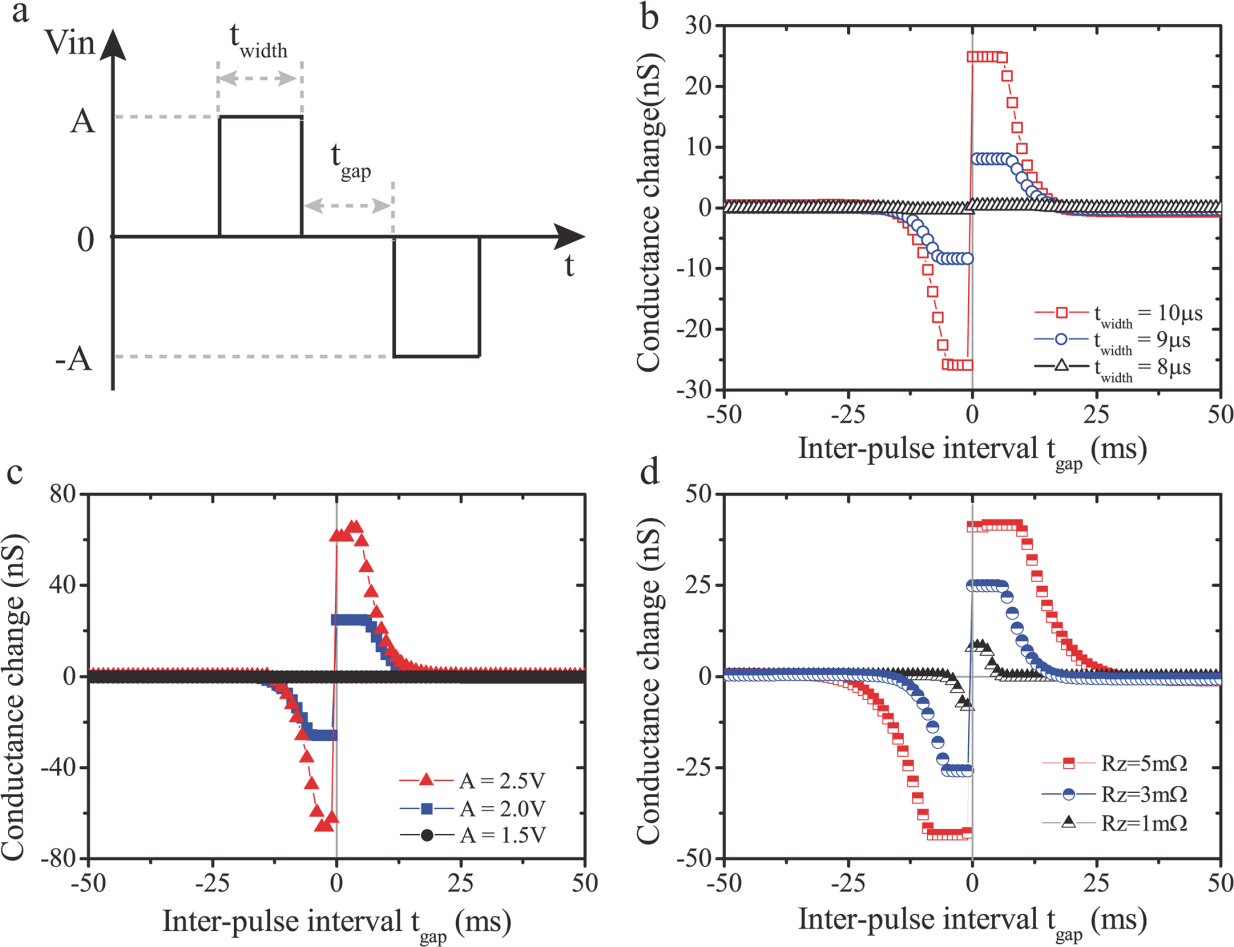
(a) Pulse pair stimulation paradigm. *A*, *t*_*width*_, and *t*_*gap*_ represent pulse magnitude, width, and inter-pulse interval. For all bipolar pulse pair-based simulations, we use *m* = 1 in function *ϕ* of module V. All other parameters were kept same as previously stated values. (b) and (c) Simulated STDP results for varying stimulus width and amplitude. (d) STDP results for varying *R*_*z*_ values indicating different decay constants of the STDP curve with increasing inter-pulse interval.

We also employed bipolar digital pulse pairs to represent spike pairs, as depicted in Fig. 4A. In each specific pair, the ‘Pre’- and ‘Post’- spikes were represented by a positive and a negative pulse respectively with magnitude *A* and width *t*_*width*_, while the inter-pulse interval (IPI) was set to *t*_*gap*_. Initially, we employed single pulse pairs whilst sweeping IPI between −50ms and 50ms in steps of 0.5ms to demonstrate the capability of our model in capturing STDP, and the dependence of STDP on pulse width and magnitude. The STDP curves shown in Fig. 4B were attained by varying pulse widths (8μs, 9μs and 10μs) at fixed a amplitude of 2V, while the results of Fig. 4C were attained by varying pulse potentials (1.5V, 2.0V and 2.5V) at a fixed pulse width of 10μs. In both cases, the conductance changes were calculated based on the initial and final values of non-volatile memristance (*v*_*y*_) in each simulation cycle. Clearly, both pulse width and magnitude significantly affect the pair-based STDP, which are attributed to the threshold switching characteristics of our model. In specific, when relatively ‘weak’ (in both pulse width and magnitude) stimuli are employed, the drive effort variable *v*_*z*_ cannot exceed the bipolar switching thresholds (*B*_+_ and *B*_−_) and thus no significant potentiation or depression is observed for all IPI values. In contrast, when the pulsing width or magnitude was increased to values where *v*_*z*_ could exceed these thresholds, good quality STDP curves could be attained to resemble the biological ones presented by Bi and Poo [12], [13]. This set of results is in agreement with experimental data captured from TiO_2_-based solid-state memristors that we have published previously in [19]. It should be noted that the peaks of the STDP curve in Fig. 4B and 4C are rather flat indicating that at short intervals, the influence of the second pulse in the pair on drive effort variable *v*_*z*_ is completely counteracted by the still-present effects of the first pulse.

We further explored the impact of driving effort module circuit parameters on STDP. Notably, the time constant of the *R*_*z*_ / *C*_*z*_ leaky integrator is given by:

$$\tau =R_{z}C_{z}$$ (8)

Fig. 4D illustrates simulated STDP curves with varying *R*_*z*_ in the driving effort module at pulse amplitude and width of 2V, 10μs respectively. It is clear that distinct *R*_*z*_ values can significantly affect both the peak amplitudes and the decay constants of the STDP curve. In case of larger *R*_*z*_ (5mΩ), drive effort leakage is limited, thus the corresponding STDP curve will attain higher STDP peak amplitude and decay more slowly as absolute IPI increases. In contrast, a smaller *R*_*z*_ (5mΩ) would accelerate leakage and result in low STDP peak amplitude and faster decay with IPI.

**Fig 5:**
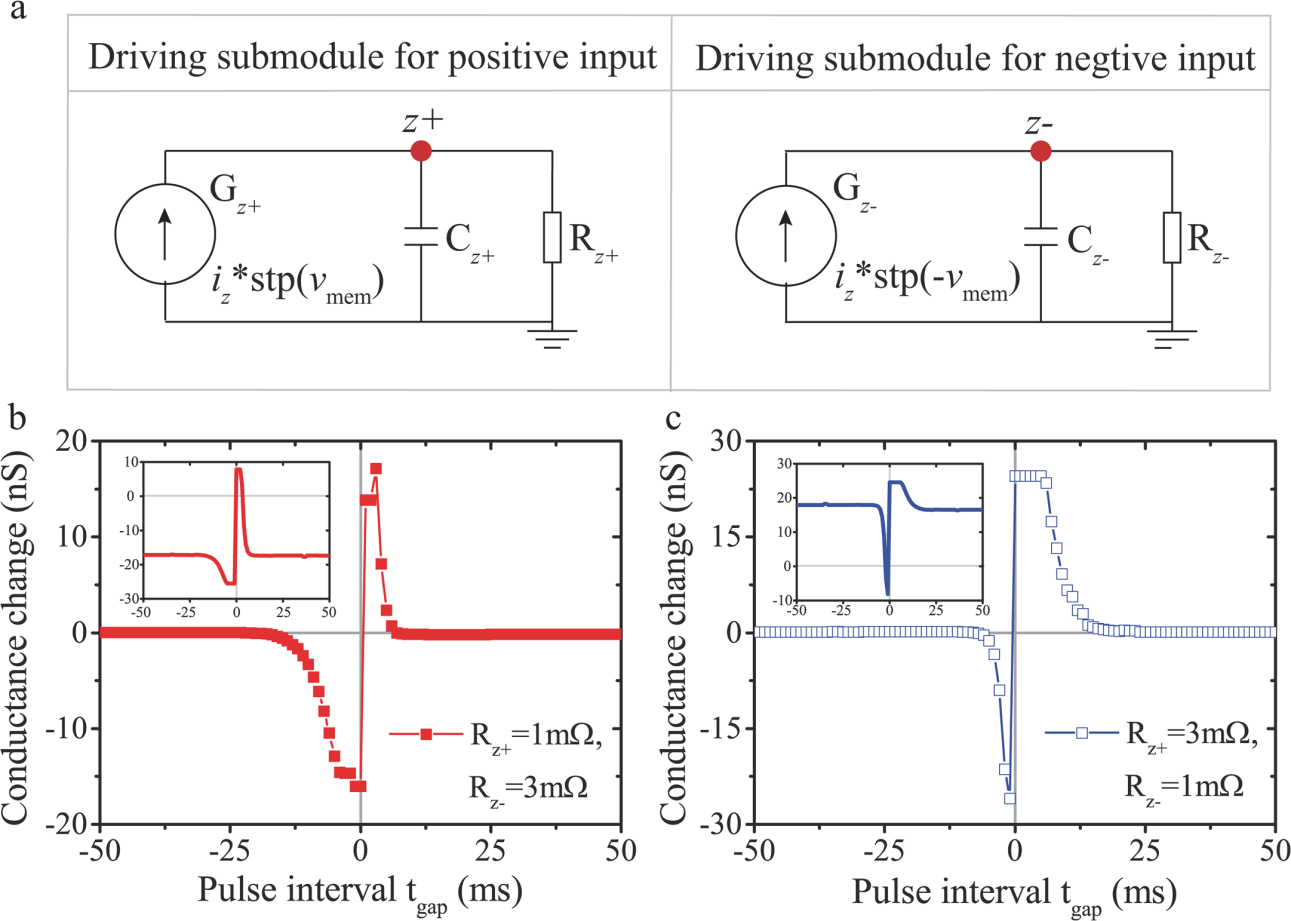
(a) Two individual driving effort sub-modules responding for only positive and only negative inputs respectively. The overall drive effort variable *v*_*z*_ equals the sum of *v*_*z*+_ and *v*_*z*−_. (b) and (c) Asymmetric STDP curves attained by employing different resistance values in the two driving sub-modules and modifying bipolar threshold values (*B*_+_ = 0.31nV, *B*_−_ = −0.27nV) to compensate for STDP curve drift. Inset: Corresponding STDP curves with original bipolar threshold values (*B*_+_ = −*B*_−_ = 0.35nV).

As a result of the construction of the driving effort module, our model tends to respond symmetrically to positive and negative pulses, which results in the symmetric STDP curves in Fig. 4. Nonetheless, it has been demonstrated that synapses possess temporally asymmetric STDP, i.e. respond distinctly to pre-post and post-pre spiking patterns [12], [13]. Moreover, the STDP curves attained from practical memristive devices are also asymmetric [20]. Therefore, we further expand this model to break the STDP symmetry by dividing the driving effort module into two individual sub-modules responding differently to opposite pulse polarities. As illustrated in Fig. 5A, two individual driving modules were set up to process positive and negative inputs separately. The overall driving effort variable *v*_*z*_ is now given by:

$$v_{z}=v_{z+}+v_{z-}$$ (9)

The STDP asymmetry arises by using different *R*_*z*_ values in the two sub-modules. Nonetheless, it should be noted that changing *R*_*z*_ values results in STDP curve drift. For example, a decrease in *R*_*z*+_ tends to shift the entire STDP curve downwards whilst a decrease in *R*_*z*−_ has the opposite effect as depicted in the inset of Fig. 5B and 5C. In our model, we compensate for STDP curve drift by optimizing the threshold for each set of *R*_*z*±_ components and input pulse specifications. Specifically, we balanced the STDP curve back to zero in Fig. 5B by setting *B*_+_ to 0.31nV, while *B*_−_ was changed to −0.27 nV in Fig. 5C. Clearly, in the former case the smaller *R*_*z*+_ (1mΩ) intensifies leakage for positive pulses only and eventually results a symmetry break of the STDP curve. A similar response is attained for smaller *R*_*z*−_ (1mΩ) in the latter case.

### 3.4. Activity dependence

**Fig 6:**
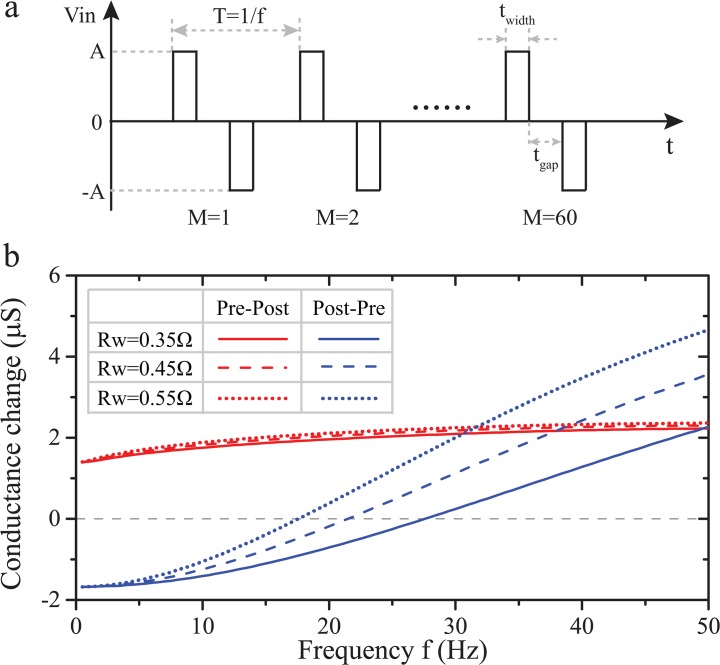
(a) Scheme of 60 repeated pulse pairs. In each pulse pair, the pulse parameters were set as A = 2V, *t*_*width*_ = 10μs, and *t*_*gap*_ = ±3ms for pre-post and post-pre pairs respectively. (b) Dependence of overall conductance modification after application of the input pulse pair train on pulse pair frequency.

We further verified the capability of our model to capture the dependence of synaptic modification on the repetition frequency of spike pair stimuli as observed in biology [14]. As depicted in Fig. 6A, 60 biphasic pulse pairs were emitted at intervals of *T* = 1 / *f*, where f is the frequency in Hz. Each pulse pair consisted of two pulses of 2V magnitude and 10μs duration, while IPI was fixed at 3ms for both post-pre- and pre-post-type stimuli. Frequency f was swept from 0.5Hz to 50Hz in steps of 0.5Hz with results illustrated in Fig. 6B. The degree of potentiation observed after the application of the stimulus is correlated to the increase of pulse pair repetition frequency for the pre-post case. In contrast, post-pre pairs result in depression at low frequencies up to 21.5Hz (for the case of *R*_*w*_ = 0.45Ω), beyond which point we obtain potentiation. The simulation results are in great agreement with the experimental data from real synapses [14]. It is worth pointing that circuit parameters *R*_*w*_ and *C*_*w*_ in the activity dependence module can be conceived as factors determining the rate of heat dispersion inside the memristor and could thus accentuate or blunt the observed frequency dependence, as demonstrated in Fig. 6B.

## Conclusion

In conclusion, we have established a new memristor SPICE model that is capable of capturing volatile and non-volatile memristance dynamics, pair-based STDP, and synaptic activity dependence. It is worth stressing that all simulations were implemented by employing simple, non-overlapping voltage pulses, which allows this model to emulate the aforementioned biological synaptic protocols on systems that use electronically convenient non-overlapping bias signals. This indicates that there is no need to build complex circuitry for generating tailor-made spike waveforms, and thus make it possible to investigate memristor based synaptic emulators and neuromorphic applications with standard digital circuitry.
